# Engineering of Yeast Old Yellow Enzyme OYE3 Enables Its Capability Discriminating of (*E*)-Citral and (*Z*)-Citral

**DOI:** 10.3390/molecules26165040

**Published:** 2021-08-20

**Authors:** Tairan Wang, Ran Wei, Yingting Feng, Lijun Jin, Yunpeng Jia, Duxia Yang, Zuonan Liang, Mengge Han, Xia Li, Chenze Lu, Xiangxian Ying

**Affiliations:** 1 Key Laboratory of Bioorganic Synthesis of Zhejiang Province, College of Biotechnology and Bioengineering, Zhejiang University of Technology, Hangzhou 310014, China; 13591715817@163.com (T.W.); weiranzjut@163.com (R.W.); fengyingting2021@163.com (Y.F.); jinlijun130@163.com (L.J.); ddbbjjpp123@163.com (Y.J.); Y1936279940@163.com (D.Y.); znznlcccc@163.com (Z.L.); a13072676126@163.com (M.H.); lixia970419@163.com (X.L.); 2 College of Life Sciences, China Jiliang University, Hangzhou 310018, China; chenzelu@cjlu.edu.cn

**Keywords:** old yellow enzyme, glucose dehydrogenase, (*R*)-citronellal, (*E*)-citral, (*Z*)-citral, asymmetric reduction, resolution

## Abstract

The importance of yeast old yellow enzymes is increasingly recognized for direct asymmetric reduction of (*E*/*Z*)-citral to (*R*)-citronellal. As one of the most performing old yellow enzymes, the enzyme OYE3 from *Saccharomyces cerevisiae* S288C exhibited complementary enantioselectivity for the reduction of (*E*)-citral and (*Z*)-citral, resulting in lower *e.e.* value of (*R*)-citronellal in the reduction of (*E*/*Z*)-citral. To develop a novel approach for the direct synthesis of enantio-pure (*R*)-citronellal from the reduction of (*E*/*Z*)-citral, the enzyme OYE3 was firstly modified by semi-rational design to improve its (*R*)-enantioselectivity. The OYE3 variants W116A and S296F showed strict (*R*)-enantioselectivity in the reduction of (*E*)-citral, and significantly reversed the (*S*)-enantioselectivity in the reduction of (*Z*)-citral. Next, the double substitution of OYE3 led to the unique variant S296F/W116G, which exhibited strict (*R*)-enantioselectivity in the reduction of (*E*)-citral and (*E*/*Z*)-citral, but was not active on (*Z*)-citral. Relying on its capability discriminating (*E*)-citral and (*Z*)-citral, a new cascade reaction catalyzed by the OYE3 variant S296F/W116G and glucose dehydrogenase was developed, providing the enantio-pure (*R*)-citronellal and the retained (*Z*)-citral after complete reduction of (*E*)-citral.

## 1. Introduction

(*R*)-citronellal serves as the key intermediate in the synthesis of L-menthol, which is one of the most popular flavors in the world [1,2,3,4]. The industrial production of (*R*)-citronellal is currently synthesized through chemical approaches starting from myrcene (Takasago company in Japan) or citral (BASF company in Germany). In the Takasago method, myrcene is converted to geranylamine, then geranylamine is isomerized and hydrolyzed to form (*R*)-citronellal [5,6]. The BASF method is simpler, in which (*E*/*Z*)-citral is distilled to obtain high-purity (*E*)-citral and then (*R*)-citronellal is synthesized through asymmetric hydrogenation of (*E*)-citral [7,8]. Chemical methods employ metal catalysts at elevated temperatures and in organic solvents, which is accompanied with undesired economic and environmental impacts. Meanwhile, biocatalysis is currently recognized as a promising alternative, considering the demands of modern organic synthesis [9,10]. Specifically, one-step synthesis of (*R*)-citronellal through enzymatic asymmetric hydrogenation of (*E*/*Z*)-citral is the most desired in the industry [8,11]. Old yellow enzymes (OYEs) are capable of catalyzing the C=C bond reduction of α,β-unsaturated compounds such as (*E*)-citral and *(Z*)-citral [12,13]. Natural (*E*/*Z*)-citral was the crude mixture of ~60% (*E*)-citral and ~40% (*Z*)-citral [14]. Unfortunately, the reduction of (*E*)-citral and *(Z*)-citral by the same native OYE usually yielded the enantio-complementary products, so the *e.e.* value of the product from the reduction of (*E*/*Z*)-citral was often relatively low [11,15].

Protein engineering has been proved to be powerful for overcoming the deficiencies of native OYEs, and successful examples of the improvement of enantioselectivity are accumulating [16,17,18,19]. Site-directed mutagenesis technique was widely used for the modification of OYEs, and various key residues determining the enantioselectivity were reported, including W116 and F296 in OYE1 [20,21,22], Y78, I113, and F247 in OYE2.6 [23]; C26, I69, and H167 in ene reductases YqjM [24]; W100 in OYE from *Gluconobacter oxydans* (Gox0502); and W66 in NCR ene reductase [25,26]. The common strategy engineering the enantioselectivity of OYEs in citral reduction was to change the substrate-binding mode, enabling one citral isomer to bind with a flipped orientation, while maintaining the orientation of the other citral isomer similar to the wild-type enzyme [17]. In old yellow enzyme OYE2y, the double substitutions of R330H/P76M, P76G/R330H, and P76S/R330H improved (*R*)-enantioselectivity to >99% *e.e.* in the reduction of (*E*)-citral or (*E*/*Z*)-citral [11]. Remarkably, those variants also enabled (*Z*)-citral to bind with a flipped orientation in the active site, and thus reversed (*S*)-enantioselectivity (32.66% *e.e.*) to (*R*)-enantioselectivity (~75% *e.e.*). To the best of our knowledge, no OYE variant was reported to reverse (*S*)-enantioselectivity to strict (*R*)-enantioselectivity (>99% *e.e.*), yet, demonstrating that direct synthesis of enatio-pure (*R*)-citronellal from the asymmetric reduction of (*E*/*Z*)-citral remained challenging. Besides the change of the substrate-binding mode, a novel strategy relying on the utilization difference of (*E*)-citral and (*Z*)-citral could be attempted to improve (*R*)-enantioselectivity. Yeast OYEs exhibited the opposite enantioselectivity in the asymmetric reduction of (*E*)-citral and (*Z*)-citral, demonstrating (*R*)-enantioselectivity and (*S*)-enantioselectivity, respectively [11]. It is reasonable to expect that the (*R*)-enantioselectivity of yeast OYEs would be increased if the utilization of (*Z*)-citral was diminished or even abolished. On the other hand, disabling the reduction of (*Z*)-citral could simultaneously lead to two isomer: enantio-pure (*R*)-citronellal from the reduction of (*E*)-citral and the retained (*Z*)-citral, both of which are valuable for perfume production and organic synthesis [27].

**Scheme 1:**
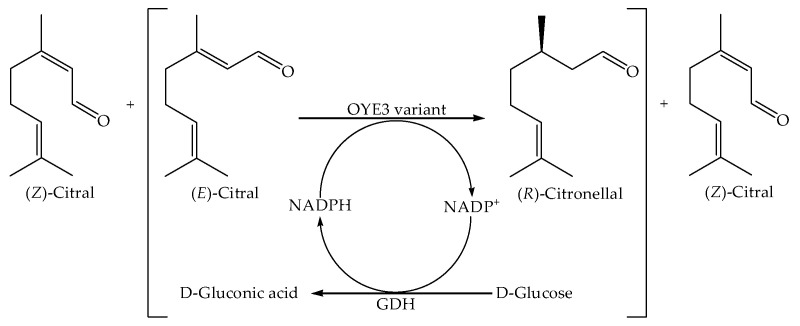
The OYE3 variant-mediated reduction of (*E/Z*)-citral to (*R*)-citronellal and the retained (*Z*)-citral with the assistance of glucose dehydrogenase-catalyzed NADPH regeneration.

In contrast to bacterial OYEs with preference of (*S*)-enantioselectivity, yeast OYEs are usually afforded to (*R*)-enantiomer in the reduction of (*E*/*Z*)-citral [28]. Yeast OYE1, OYE2, and OYE3 are the most performing members of this enzyme class [29], whereas no attempt of protein engineering has ever been reported for OYE3, implying that OYE3 has great potential of exploration. Here, aiming to develop a novel approach for the direct synthesis of enantio-pure (*R*)-citronellal from the reduction of (*E*/*Z*)-citral, the enzyme OYE3 was engineered through the modification of both the substrate-binding mode and the substrate-utilization mode. The resulting OYE3 variant discriminating (*E*)-citral and (*Z*)-citral was used to cascade asymmetric reduction of (*E*)-citral and glucose dehydrogenase (GDH)-catalyzed NADPH regeneration (Scheme 1), affording to the synthesis of enantio-pure (*R*)-citronellal and the retention of (*Z*)-citral.

## 2. Results and Discussion

### 2.1. Identification of Key Residues for the Enantioselectivity of OYE3

The enzyme OYE3 had the opposite enantioselectivity in the asymmetric reduction of (*E*)-citral and (*Z*)-citral, whose corresponding *e.e.* values were 63.45% (*R*) and 47.22% (*S*), respectively (Table 1). Thus, the *e.e.* values for the reduction of (*E*/*Z*)-citral was lower than that for the reduction of (*E*)-citral. To improve the (*R*)-enantioselectivity in the reduction of (*E*/*Z*)-citral, site-directed mutagenesis was conducted to test whether key residues critical for other OYEs determined the enantioselectivity of OYE3 [20,21,22,23,24,25,26]. OYE3 and its 12 variants were expressed in *Escherichia coli* and purified for the evaluation of catalytic performance (Figure S1). According to the change in the enantioselectivity, the variants could be classified into three groups. The variants W116A, S296F, H329R, and R38H showed improved (*R*)-enantioselectivity, whereas the variants I75C, P77C, and V113A resulted in improved (*S*)-enantioselectivity, suggesting that the enantioselectivity of OYE3 was not determined by a single amino acid residue. Besides, all the other tested variants showed similar enantioselectivity to that of OYE3. 

**Figure 1:**
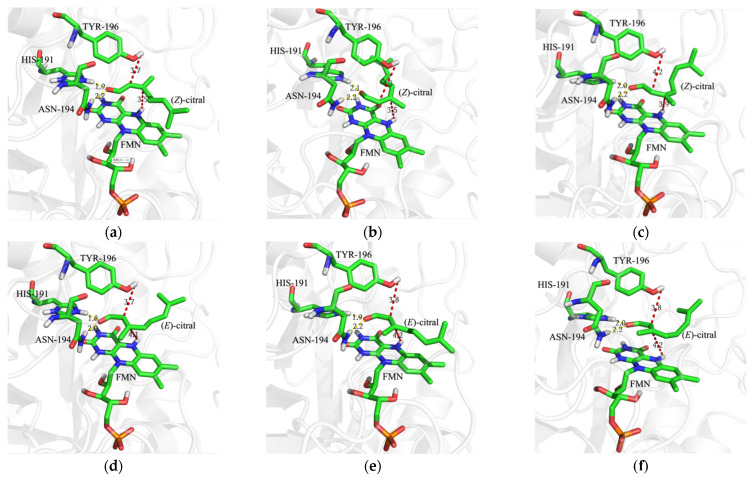
The binding modes of citral isomers in OYE3 and its variant S296F and W116G leading to either (*R*)- or (S)-citronellal. (**a**) OYE3 and (*Z*)-citral; (**b**) W116A and (*Z*)-citral, flipped binding orientation; (**c**) S296F and (*Z*)-citral, flipped binding orientation; (**d**) OYE3 and (*E*)-citral; (**e**) W116A and (*E*)-citral, preserved binding orientation; (**f**) S296F and (*E*)-citral, preserved binding orientation. Green, carbon atom; blue, nitrogen atom; tangerine, oxygen atom; white, hydrogen atom; orange, phosphorus atom.

Both the variants W116A and S296F showed strict (*R*)-enantioselectivity in the reduction of (*E*)-citral, and reversed the (*S*)-enantioselectivity in the reduction of (*Z*)-citral. The *e.e.* value in W116A-mediated reduction of (*E*/*Z*)-citral reached up to >99% despite lower conversion. To get detailed insights into the molecular mechanism, the substrate and FMNH_2_ were docked in silico into the crystal structure of OYE3 and its variants W116A and S296F. In the docking of OYE3, a conserved H191/N194 pair formed hydrogen bonds with the carbonyl oxygen of citral; a hydride was enantioselectively transferred to the substrate C_β_ atom from FMNH_2_; and the Y196 residue provided a proton to the substrate C_α_ atom as an electron acceptor [22,30]. The docking analyses suggested that the reversed enantioselectivity in the reduction of (*Z*)-citral was due to the flipped binding orientation that placed the opposite face of the alkene above the *si* face of the FMNH_2_ cofactor (Figure 1a–c). Meanwhile, the same variant reduced (*E*)-citral with preserved (*R*)-enantioselectivity derived from the same binding orientation as wild-type OYE3 (Figure 1d–f). In addition, the decreased catalytic efficiency of W116A might be attributed to the elongated distances between the substrate C_β_ atom and FMNH_2_ and the weakened hydrogen bonds between the H191/N194 pair and carbonyl oxygen of citral (Figure 1b,e). 

### 2.2. Exploration of Double Substitution at the Sites W116 and S296 of OYE3

To explore whether another substitution is superior to S296F, the residue S296 was replaced to be the amino acids with bulky side groups such as W and Y, or those with small side groups such as A and G (Table 2). As shown in Figure S2, four variants were expressed and purified to homogeneity. All the resulting variants had no improvement of (*R*)-enantioselectivity in comparison with S296F, suggesting that subtle change at the site S296 would result in significantly different enantioselectivity [31]. Similarly, W116 was tested to be V, I, F, Y, S, and G (Figure S2). As opposed to the tested S296 variants, the enantioselectivity of the resulting W116 variants showed certain correlation with the size of side group of the substituted amino acid. The variants W116F and W116Y exhibited decreased (*R*)-enantioselectivity in the reduction of (*E*)-citral, (*Z*)-citral, or (*E*/*Z*)-citral. The (*R*)-enantioselectivity of W116V and W116I fell between OYE3 and the variant W116A. In the case of W116A, the increase of available free space around W116 by the insertion of Ala, which is less hindered than Trp, seemed to favor a flipped binding mode for (*Z*)-citral and promote the (*R*)-enantioselectivity up to >99% [22]. The variants W116S and W116G showed the same strict (*R*)-enantioselectivity as W116A, but had no activity on the substrate (*Z*)-citral, suggesting that the site W116 was not only critical for the determination of enantioselectivity but also the discrimination of (*E*)- and (*Z*)-citrals.

In contrast to the S296 variants, the catalytic activities of the W116 variants were severely decreased. To investigate the effect of the double substitutions of W116 and S296 on catalytic activity, we conducted site-directed mutagenesis of S296 to F and W116 to V, S, A, and G, resulting in the four variants (Figure S3). All four resulting variants showed strict (*R*)-enantioselectivity in the reduction of (*E*)-citral, and had no catalytic activity on the reduction of (Z)-citral (Table 3). Among them, the only variant S296F/W116G was active in the (*E*/*Z*)-citral reduction, demonstrating the subtle control on the discrimination of (*E*)-citral and (*Z*)-citral. 

**Figure 2:**
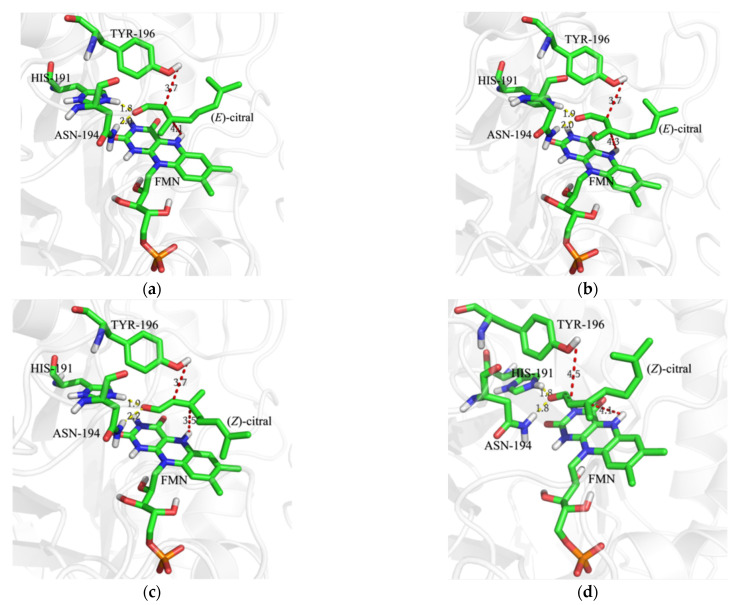
The docking analysis of the difference of OYE3 and its variant S296F/W116G on the utilization of citral isomer. (**a**) OYE3 and (*E*)-citral; (**b**) S296F/W116G and (*E*)-citral; (**c**) OYE3 and (*Z*)-citral; (**d**) S296F/W116G and (*Z*)-citral. Green, carbon atom; blue, nitrogen atom; tangerine, oxygen atom; white, hydrogen atom; orange, phosphorus atom.

The variant S296F/W116G exhibited lower catalytic efficiency than OYE3 in the reduction of (*E*/*Z*)-citral, which was supported by the determination of apparent kinetic parameters. OYE3 and its variant S296F/W116G obeyed the substrate-inhibition kinetics (Figure S4), whose inhibition might be attributed to the covalent interaction between α,β-unsaturated aldehydes and the residues of lysine and cysteine [32]. When (*E*)-citral was tested as substrate, the values of *K*_m_, *K*_i_, and *K*_cat_/*K*_m_ for the variant S296F/W116G were 0.09 mM, 5.85 mM, and 1.32 mM^−1^s^−1^, respectively (Table 4). In contrast to the variant S296F/W116G, OYE3 had lower *K*_m_ and *K*_i,_ but higher *K*_cat_/*K*_m_, implying the “trade-off” effect between (*R*)-enantioselectivity and activity. The docking analyses indicated that the accommodation of (*E*)-citral in the active site of OYE3 and the variant S296F/W116G was similar, whereas the activity loss in the variant S296F/W116G-mediated reduction of (*Z*)-citral might be attributed to the elongated distance of the hydroxyl group of W196 and the substrate C_α_ atom as well as that of the substrate C_β_ atom and FMNH_2_ (Figure 2). Since the controlling on the discrimination of (*E*)-citral and (*Z*)-citral was so subtle, crystal structures of OYE3 and the variant S296F/W116G would be required for further interpretation on its molecular mechanism.

### 2.3. Asymmetric Synthesis of (R)-Citronellal and Simultaneous Resolution of (E)-Citral and (Z)-Citral Catalyzed by the OYE3 Variant S296F/W116G and Glucose Dehydrogenase

**Figure 3:**
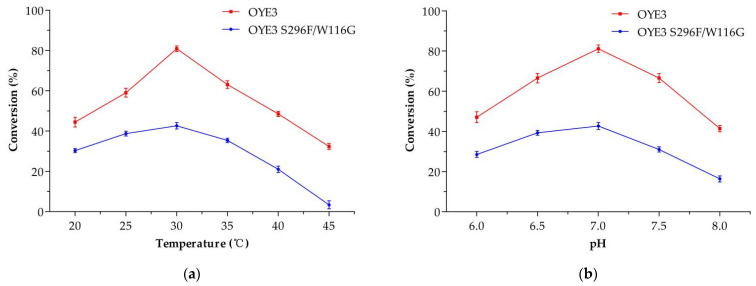
Determination of key factors affecting the asymmetric reduction of (*E/Z*)-citral to (*R*)-citronellal. The standard reaction mixture (10 mL) contained 20 mM (*E/Z*)-citral, 0.6 mM NADP^+^, 0.15 U/mL GDH, 50 mM glucose, 0.15 U/mL of the variant S296F/W116G, and 50 mM PIPES buffer solution (pH 7.0). The stock solution of substrate was 200 mM citral dissolved in isopropanol. The reaction was conducted in a reactor with a pH auto-titration system at 400 rpm for 10 h (OYE3) or 18 h (the variant S296F/W116G). The temperature was investigated from 20 to 45 °C (**a**) and the pH values were explored from 6.0 to 8.0 (**b**). Data present mean values ± SD from three independent experiments. (*E*/*Z*)-citral contained 58.4% geranial and 41.6% neral.

Both OYE3 and its variant S296F/W116G were chosen to catalyze the reduction of (*E/Z*)-citral, and the pair of GDH from *Exiguobacterium sibiricum* and glucose were used to drive the regeneration of NADPH [33]. To fulfill OYE3 variant S296F/W116G-mediate asymmetric synthesis of (*R*)-citronellal and simultaneous resolution of (*E*)-citral and (*Z*)-citral, it is mandatory to achieve complete reduction of (*E*)-citral and then cause the retention of (*Z*)-citral. In the cascade reaction, the optimization of various factors affecting the reduction of (*E*/*Z*)-citral would be significantly beneficial for the conversion improvement [34,35]. To make the results more reliable, the volume of reaction mixture was adjusted to be 10 mL and the pH was kept constant using a pH auto-titration system. During the optimization, the effect of various factors on the variant S296F/W116G was similar to that of OYE3, but lower conversion. The influence of the reaction temperature was determined over a range of 20–45 °C, and the highest conversion was observed at 30 °C (Figure 3a). When the temperature was greater than 30 °C, the conversion decreased as the temperature rose. To determine the optimal pH, the reaction was carried out at different pH values ranging from 6.0 to 8.0 at 30 °C. The highest production was detected at pH 7.0, and the conversion decreased sharply at pH values > 7.0 (Figure 3b). The enzyme activity of OYE3 requires the co-enzymes NADPH and FMNH_2_. During the cascade reaction, the enzyme-bound FMN is first reduced at the expense of NADPH, then a hydride is transferred from the reduced FMNH_2_ to the electronically activated C_β_ position of the substrate [18,22]. The in situ regeneration of NADPH is achieved by using a NAD(P)H-dependent GDH and glucose as a co-substrate. On the one hand, the supplement of exogenous NADP^+^ significantly increased the catalytic efficiency, and the NADP^+^ concentration (>0.4 mM) was required to maintain high catalytic activity (Figure S5a). On the other hand, the addition of FMN did not increase the conversion (Figure S5b), suggesting that the binding of FMN was tight enough for maintaining the activity of the variant S296/W116G [36]. In addition, the amounts of GDH and glucose were important for the efficiency of NADPH regeneration. When the substrate was 20 mM (*E/Z*)-citral, the optimized parameters for NADPH regeneration were equal activity ratio of GDH and the variant S296F/W116G (Figure S6a) and 60 mM glucose (Figure S6b).

**Figure 4:**
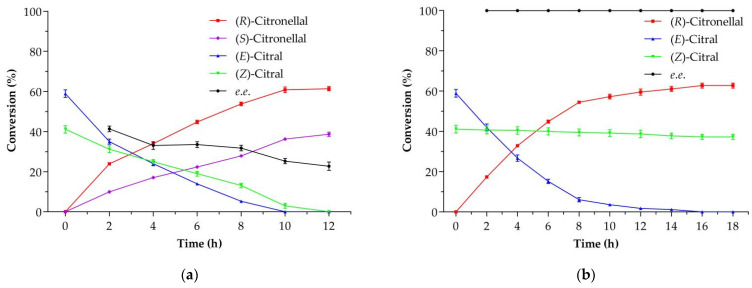
Time courses of asymmetric reduction of (*E/Z*)-citral to (*R*)-citronellal. (**a**) OYE3 and GDH as biocatalyst; (**b**) the variant OYE3 S296F/W116G and GDH as biocatalyst. The standard reaction mixture (10 mL) contained 20 mM (*E/Z*)-citral, 0.4 mM NADP^+^, 0.15 U/mL GDH, 50 mM glucose, 0.15 U/mL of the variant S296F/W116G, and 50 mM PIPES buffer solution (pH 7.0). The stock solution of substrate was 200 mM citral dissolved in isopropanol. The reaction was conducted in a reactor with a pH auto-titration system at 400 rpm and 30 °C for 12 h (OYE3) or 18 h (the variant S296F/W116G). Data present mean values ± SD from three independent experiments. (*E*/*Z*)-citral contained 58.4% geranial and 41.6% neral.

Under optimized conditions, the time course of the reduction of (*E*/*Z*)-citral catalyzed by either OYE3 or OYE3 S296F/W116G was depicted (Figure 4). In the case of OYE3-catalyzed reduction of (*E*/*Z*)-citral, the conversions to both (*R*)-citronellal and (*S*)-citronellal increased as (*E*)-citral and (*Z*)-citral were gradually utilized till complete consumption at 12 h. Since OYE3 had the complementary enantioselectivity for (*E*)-citral and (*Z*)-citral, the *e.e.* value of (*R*)-citronellal was no greater than 40%. The catalytic performance of OYE3 S296F/W116G was notably different from that of OYE3. It took 18 h for the variant OYE3 S296F/W116G to completely reduce (*E*)-citral, demonstrating its lower catalytic activity than that of OYE3. Most of (*Z*)-citral was retained and the slight decrease might be attributed to the isomerization of (*Z*)-citral to (*E*)-citral. As anticipated, (*S*)-citronellal was not detectable and the *e.e.* values of (*R*)-citronellal remained >99%, verifying that the proposed approach for asymmetric synthesis of (*R*)-citronellal and simultaneous resolution of (*E*)-citral and (*Z*)-citral was feasible.

## 3. Materials and Methods

### 3.1. Genes, Organisms, and Chemicals 

The genes encoding OYE3 and glucose dehydrogenase from *Exiguobacterium sibiricum* were codon-optimized and then synthesized by Tsingke Biotechnology Co., Ltd. (Hangzhou, China) (Figure S7). The pET28b expression vector was used for over-expression of the enzyme OYE3 and GDH, and the *E. coli* strain BL21(DE3) was used as the host. *E. coli* cultures were grown routinely in Luria Bertani (LB) medium at 37 °C for 12 h. 

(*Z*)-citral and (*E*)-citral were prepared by a previously-described procedure [11,37]. Other chemicals, including the standards, were of analytical grade and purchased from Sangon Biotech Co. Ltd. (Shanghai, China) or Sigma-Aldrich (Shanghai, China). The site-directed mutagenesis kit was obtained from Vazyme Biotech Co., Ltd. (Nanjing, China). The Ni-NTA-HP resin column for protein purification was purchased from GE Healthcare Life Sciences (Shanghai, China).

### 3.2. Over-Expression and Purification of OYE3 and GDH

The gene encoding OYE3 (Figure S7) was inserted into the sites *Nco* I/*Xho* I of the plasmid pET28b, resulting in the plasmid pET28b-*oye3*. The plasmid pET28b-*oye3* was transformed into *E. coli* BL21 (DE3) competent cells, resulting in the strain *E. coli* BL21(DE3)/pET28b-*oye3*. The cells containing pET28b-*oye3* were cultured in the LB medium with 100 µg/mL kanamycin at 37 °C and 200 rpm until the OD_600_ of 0.6, and 0.2 mM isopropyl β-D-1-thiogalactopyranoside (IPTG) was supplemented for the induction at 25 °C and 160 rpm. After 12 h of induction, *E. coli* cells were harvested by centrifugation and further washed using 50 mM Tris-HCl buffer (pH 8.0). The productivity of OYE3 was 2.42 U per gram of wet cells. The cells were disrupted through ultrasonication for 10 min, and the cell debris was removed by centrifugation to result in a clear cell extract. The crude cell extracts containing OYE3 was applied to a Ni-NTA chelating affinity column equilibrated with the binding buffer (5 mM imidazole and 300 mM NaCl dissolved in 50 mM Tris-HCl, pH 8.0). Unbound proteins were washed off by the application of the binding buffer. The OYE3 was eluted with 100 mM imidazole in 50 mM Tris-HCl (pH 8.0), desalted with 50 mM Tris-HCl buffer (pH 8.0) by ultrafiltration, and then stored at −20 °C for further study.

The gene encoding GDH (Figure S7) was inserted into the sites *Nde* I/*Xho* I of the plasmid pET28b, resulting in the plasmid pET28b-*gdh*. Following the similar procedure to OYE3, the cells harboring pET28b-*gdh* was induced and the enzyme GDH was purified for further use. The purity of the purified enzymes was visualized through SDS-PAGE analysis using a 12% resolving gel [38].

### 3.3. Construction of OYE3 Variants by Site-Directed Mutagenesis

The site-directed mutagenesis was performed as previously described [11]. PCR amplification to introduce substitution was performed in 30 µL of standard PCR mixture with 50 ng of template plasmid DNA and 15 pmol each of the appropriate set of primers using the following temperature cycle; 5 min at 95 °C, followed by 30 cycles of 95 °C for 15 s, appropriate annealing temperature (55–61 °C) for 15 s, and 72 °C for 1 min, and the final extension of 5 min at 72 °C. The plasmid pET28b-*oye3* was used as template DNA in the single substitution, while the creation of double substitutions was based on the gene encoding the variant OYE3 S296F as the template. The primer sets for single or double substitutions were shown in Tables S1–S3. The amplified PCR fragments were digested with the restriction enzyme *Dpn* I at 37 °C for 1 h, and then the digested DNA was directly introduced into *E. coli* strain BL21(DE3). Each constructed plasmid was confirmed by sequencing. Expression and purification of the resulting OYE3 variants were conducted using the same procedure as OYE3. Specifically, the productivity of OYE3 S296F/W116G was 1.74 U per gram of wet cells. 

### 3.4. Assays of Glucose Dehydrogenase and Old Yellow Enzymes

Activities of the purified OYEs and GDH were measured at 30 °C by monitoring the change of the absorbance at 340 nm. The enzyme assay for OYEs was carried out in triplicate in a reaction mixture (1 mL) composed of 2 mM (*E*)-citral and 0.2 mM NADPH in 100 mM PIPES buffer (pH 7.0). The enzyme assay for GDH was carried out in triplicate in a reaction mixture (1 mL) composed of 5 mM glucose and 0.4 mM NADP^+^ in 100 mM PIPES buffer (pH 7.0). Both reactions were started by the addition of the enzyme (1 μg for GDH, 5 μg for OYE3 or its variants). One unit of the activity is defined as formation or oxidation of 1 μmol NADPH per min. The protein concentrations of all samples were determined using the BCA reagent with bovine serum albumin as the standard protein [39]. 

Apparent kinetic parameters were determined using different concentrations of (*E*)-citral and (*Z*)-citral. Concentrations of (*E*)-citral and (*Z*)-citral were 0.01, 0.05, 0.1, 0.5, 1, 2, 5, 10, 20, and 50 mM. According to substrate-inhibition kinetics, apparent values of *K*_m_, *K*_i_, and *V*_max_ were calculated using the curve fittings of the software Prism 7.2 (GraphPad Software, San Diego, CA, USA).

### 3.5. Homology Modeling and Molecular Docking

Using the crystal structure of OYE3 from *S. cerevisiae* S288C (PDB number: 5v4p, resolution 1.88 Å), molecular docking simulation was performed using AutoDock Vina under the default docking parameters [40]. Point charges were initially assigned according to the AMBER03 force field and then damped to mimic the less polar Gasteiger charges [41]. (*E*)-citral or (*Z*)-citral acted as a ligand to molecular docking with OYE3, and the calculation of geometric parameters and ligand structure was performed by ChemBioDraw 12.0 (CambridgeSoft, Cambridge, MA, USA). All homology models of OYE3 variants were built using the “BuildModel” tool implemented in FoldX software with the model of OYE3 as a template [42,43,44]. To make the results more accurate, 20 consecutive runs were performed and the highest ranked score from each run was used to calculate the average score of each flexible ligand configuration. The docking result with the minimal binding energy value was selected from 100 predictions. The visualization of the resulting substrate–enzyme complexes was processed using the PyMol software [45].

### 3.6. Investigation of Catalytic Performance of OYE3 and Its Variants in the Reduction of (E)-, (Z)-, or (E/Z)-Citral

To determine catalytic performance of OY3 and its variants, the standard reaction mixture (1 mL) contained 20 mM citral, 0.6 mM NADP^+^, 0.15 U/mL GDH, 50 mM glucose, 0.15 U/mL OYE3 or its variant, and 50 mM PIPES buffer solution (pH 7.0). The substrate included (*E*)-citral, (*Z*)-citral, or (*E*/*Z*)-citral, whose stock solution was 200 mM substrate in isopropanol. The reaction mixture was incubated in an orbital shaker (200 rpm, 30 °C) for 11 h, and then extracted using an equal volume of ethyl acetate at 30 °C and 200 rpm for 2 h. After extraction and centrifugation (12,000 rpm, 1 min), the solvent phase was collected, dried over anhydrous sodium sulfate, and subjected to GC analyses as previously described [11]. All reactions were carried out in triplicate. 

The GC analyses of substrates and products were conducted using the GC (Agilent 6890N) equipped with an FID detector and chiral capillary BGB-174 column (BGB Analytik, Switzerland, 30 m length, 250 μm inner diameter, 0.25 μm film thickness). The flow rate and split ratio of N_2_ as the carrier gas were set as 2 mL/min and 1:49, respectively. The temperatures for both injector and detector were set as 250 °C. The column temperature program was listed as follows: initial temperature of 90 °C for 25 min, 20 °C/min ramp to 160 °C for 2 min, and 20 °C/min ramp to 180 °C for 3 min. The injection volume was 1 μL. The retention times of (*S*)-citronellal, (*R*)-citronellal, (Z)-citral and (*E*)-citral were 22.5, 23.0, 29.2, and 30.2 min (Figure S8). 

### 3.7. Identification of Key Factors for S296F/W116G Mediated-Reduction of (E)-Citral

To identify key factors for S296F/W116G mediated-reduction of (*E/Z*)-Citral, the standard reaction mixture (10 mL) contained 20 mM (*E/Z*)-citral, 0.6 mM NADP^+^, 0.15 U/mL GDH, 50 mM glucose, 0.15 U/mL the variant S296F/W116G, and 50 mM PIPES buffer solution (pH 7.0). The reaction was conducted at 400 rpm and 30 °C for 10 h (OYE3) or 18 h (the variant S296F/W116G) in a reactor with pH-constant auto-titration system. The solution for pH auto-titration was 1 M NaOH. The optimal temperature of (*E*)-citral reduction was determined at a series of temperatures ranging from 20 to 45 °C. The optimal pH was determined over a range of pH 6.0 to 8.0 at 30 °C. The concentrations of NADP^+^ were explored within the range of 0 to 1 mM. The optimal glucose concentration was determined over a range of 1 to 100 mM. The activity ratio of GDH and the variant S296F/W116G was tested to be 1:4, 2:4, 4:4, 6:4, or 8:4. After optimization, the time courses OYE3 and the variant OYE3 S296F/W116G-mediated reduction of (*E*/*Z*)-citral were investigated in the reaction mixture as below: 20 mM (*E/Z*)-citral, 0.4 mM NADP^+^, 0.15 U/mL GDH, 60 mM glucose, 0.15 U/mL OYE3, or the variant S296F/W116G, and 50 mM PIPES buffer solution (pH 7.0). The reactions for OYE3 and the variant OYE3 S296F/W116G mediated reduction of (*E*/*Z*)-citral were conducted at 400 rpm and 30 °C for 12 h and 18 h, respectively. The reaction mixture was sampled every 2 h. The samples were extracted using an equal volume of ethyl acetate at 30 °C and 200 rpm for 2 h. Then, the solvent phase was collected, dried over anhydrous sodium sulfate, and subjected to GC analyses as described in the Section 3.6. All reactions were carried out in triplicate.

## 4. Conclusions

The enzyme OYE3 was engineered through semi-rational design to improve (*R*)-enantioselectivity in the reduction of (*E*/*Z*)-citral to (*R*)-citronellal. The substitution of S296 to F resulted in the remarkable improvement of (*R*)-enantioselectivity. The variant W116A showed strict (*R*)-enantioselectivity in the reduction of (*E*)-citral, (*Z*)-citral or (*E*/*Z*)-citral. To the best of our knowledge, this is the first report to completely reverse (*S*)-enantioselectivity to (*R*)-enantioselectivity in the OYE family. Interestingly, the further double substitution variant W116A/S296F was capable of discriminating (*E*)-citral and (Z)-citral, indicating that the sites W116 and S296 were pivotal for not only the determination of enantioselectivity, but also the discrimination of citral isomers. Using the variant OYE3 S296F/W116G and GDH as biocatalyst, a novel cascade reaction was achieved to offer two isomers in the (*E*/*Z*)-citral reduction: enantio-pure (*R*)-citronellal and the retained (*Z*)-citral after complete consumption of (*E*)-citral. 

## Figures and Tables

**Table 1:** The catalytic performances of OYE3 and its variants with single substitution at putative key residues ^1^.

Enzyme	(*E*)-Citral		(*Z*)-Citral		(*E*/*Z*)-Citral	
	*e.e.* (%)	Conversion (%)	*e.e.* (%)	Conversion (%)	*e.e.* (%)	Conversion (%)
OYE3	63 ± 1.1 (*R*)	91.9 ± 3.0	47 ± 1.2 (*S*)	88.8 ± 2.1	23 ± 1.0 (*R*)	86.6 ± 1.7
W116A	>99 (*R*)	31.9 ± 1.2	43 ± 1.0 (*R*)	7.91 ± 0.1	>99 (*R*)	9.8 ± 0.2
S296F	>99 (*R*)	71.1 ± 2.0	52 ± 0.9 (*R*)	80.1 ± 1.1	72 ± 1.5 (*R*)	80.3 ± 1.8
H329R	56 ± 0.9 (*R*)	87.9 ± 2.1	32 ± 0.7 (*S*)	86.5 ± 2.3	48 ± 0.9 (*R*)	60.4 ± 1.3
R38H	55 ± 0.8 (*R*)	89.3 ± 2.4	15 ± 0.6 (*S*)	79.4 ± 1.8	38 ± 1.2 (*R*)	69.2 ± 1.7
M67L	63 ± 1.3 (*R*)	91.5 ± 2.8	37 ± 1.1 (*S*)	89.0 ± 2.3	24 ± 0.8 (*R*)	88.8 ± 2.4
A79S	43 ± 1.0 (*R*)	88.5 ± 1.9	49 ± 1.2 (*S*)	85.7 ± 2.5	22 ± 1.3 (*R*)	86.6 ± 2.3
V288A	70 ± 1.9 (*R*)	86.2 ± 1.8	38 ± 1.3 (*S*)	84.9 ± 2.5	21 ± 1.2 (*R*)	85.6 ± 1.6
R12L	63 ± 1.2 (*R*)	94.1 ± 2.1	45 ± 1.4 (*S*)	89.4 ± 1.8	21 ± 0.8 (*R*)	92.8 ± 1.9
I344K	61 ± 1.5 (*R*)	91.7 ± 1.7	44 ± 0.8 (*S*)	94.0 ± 1.3	20 ± 0.7 (*R*)	88.6 ± 1.4
I75C	62 ± 2.1 (*R*)	91.8 ± 1.6	57 ± 1.6 (*S*)	85.9 ± 2.0	14 ± 1.0 (*R*)	92.9 ± 2.4
P77C	62 ± 1.1 (*R*)	90.3 ± 2.4	77 ± 1.7 (*S*)	96.7 ± 1.3	10 ± 0.6 (*R*)	91.8 ± 2.6
V113A	60 ± 1.6 (*R*)	97.2 ± 1.1	28 ± 0.7 (*S*)	78.8 ± 1.5	10 ± 0.8 (*R*)	89.3 ± 1.0

^1^ The reaction mixture (1 mL) contained 20 mM citral, 0.6 mM NADP^+^, 0.15 U/mL GDH, 50 mM glucose, 0.15 U/mL OYE3 or its variant, and 50 mM PIPES buffer solution (pH 7.0). The stock solution of substrate was 200 mM citral dissolved in isopropanol. The reaction was conducted in an orbital shaker (200 rpm, 30 °C) for 11 h. Data present mean values ± SD from three independent experiments. (*E*)-Citral contained 97.6% geranial and 2.4% neral, (*Z*)-citral contained 98.6% neral and 1.4% geranial, and (*E*/*Z*)-citral contained 58.4% geranial and 41.6% neral.

**Table 2:** The catalytic performances of OYE3 and its S296X and W116X variants ^1^.

Enzyme	(*E*)-Citral		(*Z*)-Citral		(*E*/*Z*)-Citral	
	*e.e.* (%)	Conversion (%)	*e.e.* (%)	Conversion (%)	*e.e.* (%)	Conversion (%)
OYE3	63 ± 1.1 (*R*)	91.9 ± 3.0	47 ± 1.2 (*S*)	88.8 ± 2.1	23 ± 1.0 (*R*)	86.8 ± 1.7
S296F	>99 (*R*)	71.1 ± 1.2	52 ± 0.8 (*R*)	80.1 ± 2.7	72 ± 1.8 (*R*)	80.3 ± 1.9
S296W	56 ± 1.0 (*R*)	81.6 ± 1.7	1.2 ± 0.5 (*R*)	82.6 ± 1.9	32 ± 2.3 (*R*)	75.6 ± 0.8
S296Y	58 ± 1.3 (*R*)	75.1 ± 1.3	41 ± 1.4 (*S*)	82.8 ± 1.4	22 ± 0.9 (*R*)	88.5 ± 1.4
S296A	54 ± 0.8 (*R*)	94.3 ± 2.0	49 ± 1.7 (*S*)	88.5 ± 1.2	15 ± 1.4 (*R*)	89.5 ± 2.3
S296G	51 ± 0.7 (*R*)	95.6 ± 2.3	44 ± 1.1 (*S*)	71.6 ± 0.9	12 ± 1.5 (*R*)	91.1 ± 2.0
W116A	>99 (*R*)	4.1 ± 0.1	>99 (*R*)	2.9 ± 0.1	>99 (*R*)	8.4 ± 0.1
W116V	76 ± 1.3 (*R*)	22.5 ± 0.4	57 ± 2.0 (*R*)	7.1 ± 0.2	>99 (*R*)	1.4 ± 0.1
W116I	47 ± 1.2 (*R*)	29.0 ± 0.7	21 ± 1.2 (*S*)	15.6 ± 0.7	44 ± 1.8 (*R*)	24.3 ± 0.5
W116F	47 ± 1.2 (*R*)	88.0 ± 2.2	67 ± 0.9 (*S*)	14.7 ± 0.4	1.7 ± 0.8 (*R*)	67.5 ± 1.4
W116Y	31 ± 1.0 (*R*)	80.8 ± 1.7	71 ± 1.5 (*S*)	65.1 ± 1.9	5.2 ± 1.0 (*S*)	57.1 ± 1.6
W116S	>99 (*R*)	14.7 ± 0.3	/	/	>99 (*R*)	9.0 ± 0.2
W116G	>99 (*R*)	11.1 ± 0.2	/	/	/	/

^1^ X represents one of the amino acids F, W, Y, A, G, or A, V, I, F, Y, S, and G. The reaction mixture (1 mL) contained 20 mM citral, 0.6 mM NADP^+^, 0.15 U/mL GDH, 50 mM glucose, 0.15 U/mL OYE3 or its variant, and 50 mM PIPES buffer solution (pH 7.0). The stock solution of substrate was 200 mM citral dissolved in isopropanol. The reaction was conducted in an orbital shaker (200 rpm, 30 °C) for 11 h. Data present mean values ± SD from three independent experiments. (*E*)-Citral contained 97.6% geranial and 2.4% neral, (*Z*)-citral contained 98.6% neral and 1.4% geranial, and (*E*/*Z*)-citral contained 58.4% geranial and 41.6% neral.

**Table 3:** The catalytic performances of the S296F/W116X variants of OYE3 ^1^.

Enzyme	(*E*)-Citral		(*Z*)-Citral		(*E*/*Z*)-Citral	
	*e.e.* (%)	Conversion (%)	*e.e.* (%)	Conversion (%)	*e.e.* (%)	Conversion (%)
S296F/W116V	>99 (*R*)	11.8 ± 0.2	/	/	/	/
S296F/W116S	>99 (*R*)	29.3 ± 1.0	/	/	/	/
S296F/W116A	>99 (*R*)	8.0 ± 0.1	/	/	/	/
S296F/W116G	>99 (*R*)	31.8 ± 0.9	/	/	>99 (*R*)	25.3 ± 0.5

^1^ X represents one of the amino acids A, V, S, and G. The reaction mixture (1 mL) contained 20 mM citral, 0.6 mM NADP^+^, 0.15 U/mL GDH, 50 mM glucose, 0.15 U/mL OYE3 or its variant, and 50 mM PIPES buffer solution (pH 7.0). The stock solution of substrate was 200 mM citral dissolved in isopropanol. The reaction was conducted in an orbital shaker (200 rpm, 30 °C) for 11 h. Data present mean values ± SD from three independent experiments. (*E*)-Citral contained 97.6% geranial and 2.4% neral, (*Z*)-citral contained 98.6% neral and 1.4% geranial, and (*E*/*Z*)-citral contained 58.4% geranial and 41.6% neral.

**Table 4:** Apparent kinetic parameters of OYE3 and its variant S296F/W116G ^1^.

Parameter	OYE3		OYE3 S296F/W116G	
	(*E*)-Citral	(*Z*)-Citral	(*E*)-Citral	(*Z*)-Citral
*K*_m_ (mM)	0.05 ± 0.01	0.07 ± 0.01	0.09 ± 0.01	/
*K*_i_ (mM)	4.53 ± 0.28	5.75 ± 0.37	5.85 ± 0.33	/
*V*_max_ (U/mg)	0.16 ± 0.03	0.12 ± 0.02	0.14 ± 0.02	/
*K*_cat_ (s^−^^1^)	0.13 ± 0.02	0.10 ± 0.01	0.11 ± 0.01	/
*K*_cat_/*K*_m_ (mM^−^^1^s^−^^1^)	2.76 ± 0.11	1.49 ± 0.08	1.32 ± 0.09	/

^1^ Data present mean values ± SD from three independent experiments.

## Supplementary Materials

The following are available online, Table S1: The primer information of site-directed mutagenesis of OYE3, Table S2: The primer information of site-directed mutagenesis of W116 and S296 in OYE3, Table S3: The primer information of combinatorial mutagenesis of W116 and S296 in OYE3, Figure S1: SDS-PAGE (12%) analysis of OYE3 and 12 purified OYE3 variants, Figure S2: SDS-PAGE (12%) analysis of OYE3 and 12 purified OYE3 variants with single substitution at site W116 or S296, Figure S3: SDS-PAGE (12%) analysis of 4 purified OYE3 variants with double substitution at sites W116 and S296, Figure S4: Apparent kinetic parameter determination of OYE3 and its variant S296F/W116G, Figure S5: Effect of NADP^+^ and FMN on the reduction of (*E*/*Z*)-citral to (*R*)-citronellal, Figure S6: Effect of glucose dehydrgenase (a) and glucose (b) on the reduction of (*E*/*Z*)-citral to (*R*)-citronellal, Figure S7: The codon-optimized nucleotide sequences encoding old yellow enzyme OYE3 from *Saccharomyces cerevisiae* S288C and glucose dehydrogenase from *Exiguobacterium sibiricum*, Figure S8: The GC chromatogram of standard substrates (a) and standard products (b).

## Sample Availability

Samples of the compounds are not available from the authors.
