# Supplementary material for: Engineering of Yeast Old Yellow Enzyme OYE3 Enables Its Capability Discriminating of (E)-Citral and (Z)-Citral
Source: Molecules. 2021 Aug 20;26(16):5040. doi: 10.3390/molecules26165040 (PMC8399149; doi:10.3390/molecules26165040)
Supplement: Supplementary file 1 [file molecules-26-05040-s001.zip › molecules-1327221-supplementary.pdf]

Supporting Information

Engineering of Yeast Old Yellow Enzyme OYE3 Enables Its Capability Discriminating of (*E*)- and (*Z*)-Citrals

Tairan Wang [1,†], Ran Wei [1,†], Yingting Feng [1], Lijun Jin [1], Yunpeng Jia [1], Duxia Yang [1], Zuonan Liang [1], Mengge Han [1], Xia Li [1], Chenze Lu [2] and Xiangxian Ying [1,*]

[1] Key Laboratory of Bioorganic Synthesis of Zhejiang Province, College of Biotechnology and Bioengineering, Zhejiang University of Technology, Hangzhou 310014, China; 13591715817@163.com (T.W.); weiranzjut@163.com (R.W.); fengyingting2021@163.com (Y.F.); jinlijun130@163.com (L.J.); ddbbjjpp123@163.com (Y.J.); Y1936279940@163.com (D.Y.); znznlcccc@163.com (Z.L.); a13072676126@163.com (M.H.); lixia970419@163.com (X.L.);

[2] College of Life Sciences, China Jiliang University, Hangzhou 310018, China; chenzelu@cjlu.edu.cn

* Correspondence: yingxx@zjut.edu.cn; Tel.: +86-571-88320781

† These authors contributed equally to this work.

Contents

.... 1

Supplementary tables.......... 2

Table S1. The primer information of site-directed mutagenesis of OYE3 [a] 2

Table S2. The primer information of site-directed mutagenesis of W116 and S296 in OYE3 [a] 3

Table S3. The primer information of combinatorial mutagenesis of W116 and S296 in OYE3 [a] 4

Supplementary figures.......... 5

Figure S1. SDS-PAGE (12%) analysis of OYE3 and 12 purified OYE3 variants. 5

Figure S2. SDS-PAGE (12%) analysis of OYE3 and 12 purified OYE3 variants with single substitution at site W116 or S296. 6

Figure S3. SDS-PAGE (12%) analysis of 4 purified OYE3 variants with double substitution at sites W116 and S296. 7

Figure S4. Apparent kinetic parameter determination of OYE3 and its variant S296F/W116G. 8

Figure S5. Effect of $NADP^+$ (a) and FMN (b) on the reduction of (*E*/*Z*)-citral to (*R*)-citronellal. 9

Figure S6. Effect of glucose dehydrgenase (a) and glucose (b) on the reduction of (*E*/*Z*)-citral to (*R*)-citronellal. 10

Figure S7. The codon-optimized nucleotide sequences encoding old yellow enzyme OYE3 from *Saccharomyces cerevisiae* S288C and glucose dehydrogenase from *Exiguobacterium sibiricum*. 11

Figure S8. The GC chromatogram of standard substrates (a) and standard products (b). 12

Supplementary tables

Table S1. The primer information of site-directed mutagenesis of OYE3 [a]

Primer		Sequence
R12L	F	5’-AGCCTGCTTGACACCAACCTGTTCGAACCGA
	R	5’-GGTGTCAAGCAGGCTAATCGGTTCAAACCCTT
R38H	F	5’-CTGACCCATATGCGTGCAACCCACCCGGGTA
	R	5’-ACGCATATGGGTCAGCGGCGGCATAACGGCA
M67L	F	5’-CTCTTATTATTACAGAGGGCACATTTATTTCACCGCAGGC
	R	5’-TAAGAGTACCCGGACGCTGTGCGCGCTGACCATAATAAA
I75C	F	5’-GGCACATTTTGTTCACCGCAGGCAGGAGGCTATGATAAT
	R	5’-CGGTGAACAAAATGTGCCCTCTGTAATAATCATAGTACC
P77C	F	5’-TTTATTTCATGTCAGGCAGGAGGCTATGATAAT
	R	5’-TGCCTGACATGAAATAAATGTGCCCTCTGTAATAATC
A79S	F	5’-TCACCGCAGTCTGGAGGCTATGATAATGCGCCGGGTATC
	R	5’-GCCTCCAGACTGCGGTGAAATAAATGTGCCCTCTGT
V113A	F	5’-GCATGGGCCCAGCTGTGGAGTCTGGGTTGGGCGAGTTTT
	R	5’-CAGCTGGGCCCATGCAAAGCTCTGACAGTCGTGAATTGC
W116A	F	5’-GTCCAGCTGGTTAGTCTGGGTTGGGCGAGTTTTCCGGAT
	R	5’-CAGACTAACCAGCTGGACCCATGCAAAGCTCTGACAGTC
V288A	F	5’-CATCTGGCTGAACCGCGTGTTACCGATCCGAG
	R	5’-CGGTTCAGCCAGATGAACAAATGCCAGACGTT
S296F	F	5’-ACGATCCGTTCCTGGTTGAAGGTGAAGGTGAATATAGCG
	R	5’-AACCAGGAACGGATCGGTAACACGCGGTTCAACCAGATG
H329R	F	5’-TATGCACTGCGTCCGGAAGTTGTTCGTGAACAGGTTAAA
	R	5’-TTCCGGACGCAGTGCATAATTACCTGCACGAATAATCGG
I344K	F	5’-AAAGATCCGCGTACCCTGAAAGGTTATGGTCGT
	R	5’-TTTCAGGGTACGCGGATCTTTAACCTGTTCACGA

[a] The code to introduce the substitution was underlined.

Table S2. The primer information of site-directed mutagenesis of W116 and S296 in OYE3 [a]

Primer		Sequence
W116F	F	5'-GTCCAGCTGTTTAGTCTGGGTTGGGCGAGTTTTCCGGAT
	R	5'-CAGACTAAACAGCTGGACCCATGCAAAGCTCTGACAGTC
W116A	F	5'-GTCCAGCTGGTTAGTCTGGGTTGGGCGAGTTTTCCGGAT
	R	5'-CAGACTAACCAGCTGGACCCATGCAAAGCTCTGACAGTC
W116Y	F	5'-GTCCAGCTGTATAGTCTGGGTTGGGCGAGTTTTCCGGAT
	R	5'-CAGACTATACAGCTGGACCCATGCAAAGCTCTGACAGTC
W116I	F	5'-GTCCAGCTGATTAGTCTGGGTTGGGCGAGTTTTCCGGAT
	R	5'-CAGACTAATCAGCTGGACCCATGCAAAGCTCTGACAGTC
W116G	F	5'-GTCCAGCTGGGTAGTCTGGGTTGGGCGAGTTTTCCGGAT
	R	5'-CAGACTACCCAGCTGGACCCATGCAAAGCTCTGACAGTC
W116V	F	5'-GTCCAGCTGGTTAGTCTGGGTTGGGCGAGTTTTCCGGAT
	R	5'-CAGACTAACCAGCTGGACCCATGCAAAGCTCTGACAGTC
W116S	F	5'-GTCCAGCTGTCTAGTCTGGGTTGGGCGAGTTTTCCGGAT
	R	5'-CAGACTAGACAGCTGGACCCATGCAAAGCTCTGACAGTC
S296F	F	5'-ACGATCCGTTCCTGGTTGAAGGTGAAGGTGAATATAGCG
	R	5'-AACCAGGAACGGATCGGTAACACGCGGTTCAACCAGATG
S296Y	F	5'-ACGATCCGTATCTGGTTGAAGGTGAAGGTGAATATAGCG
	R	5'-AACCAGATACGGATCGGTAACACGCGGTTCAACCAGATG
S296W	F	5'-ACGATCCGTGGCTGGTTGAAGGTGAAGGTGAATATAGCG
	R	5'-AACCAGCCACGGATCGGTAACACGCGGTTCAACCAGATG
S296A	F	5'-ACGATCCGGCTCTGGTTGAAGGTGAAGGTGAATATAGCG
	R	5'-AACCAGAGCCGGATCGGTAACACGCGGTTCAACCAGATG
S296G	F	5'-ACGATCCGGGTCTGGTTGAAGGTGAAGGTGAATATAGCG
	R	5'-AACCAGACCCGGATCGGTAACACGCGGTTCAACCAGATG

[a] The code to introduce the substitution was underlined.

Table S3. The primer information of combinatorial mutagenesis of W116 and S296 in OYE3 [a]

Primer		Sequence
W116A	F	5'-GTCCAGCTGGTTAGTCTGGGTTGGGCGAGTTTTCCGGAT
	R	5'-CAGACTAACCAGCTGGACCCATGCAAAGCTCTGACAGTC
W116G	F	5'-GTCCAGCTGGGTAGTCTGGGTTGGGCGAGTTTTCCGGAT
	R	5'-CAGACTACCCAGCTGGACCCATGCAAAGCTCTGACAGTC
W116S	F	5'-GTCCAGCTGTCTAGTCTGGGTTGGGCGAGTTTTCCGGAT
	R	5'-CAGACTAGACAGCTGGACCCATGCAAAGCTCTGACAGTC
W116V	F	5'-GTCCAGCTGGTTAGTCTGGGTTGGGCGAGTTTTCCGGAT
	R	5'-CAGACTAACCAGCTGGACCCATGCAAAGCTCTGACAGTC

[a] The code to introduce the substitution was underlined.

Supplementary figures

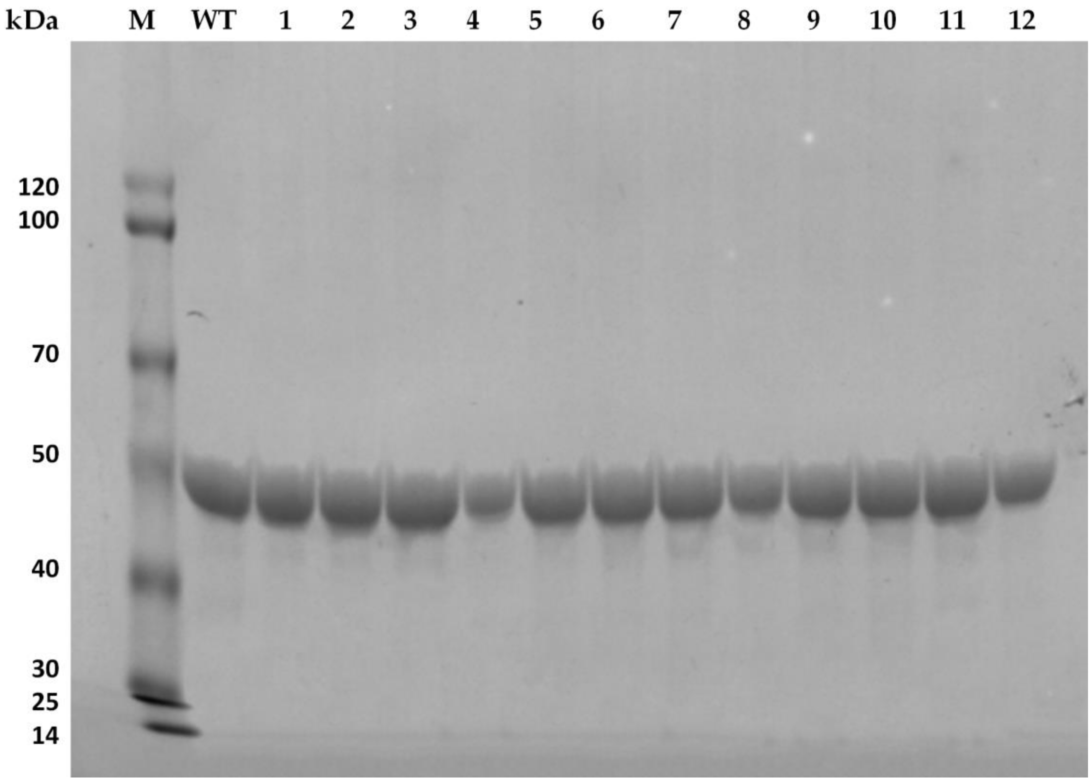

Figure S1. SDS-PAGE (12%) analysis of OYE3 and 12 purified OYE3 variants. Lane M, standard molecular mass proteins; lane WT, wild type OYE3. Other lanes from 1 to 12 represent the OYE3 variants (from left to right): R12L, R38H, M67L, I75C, P77C, A79S, V113A, W116A, V288A, S296F, H329R and I344K. The proteins were visualized by staining with Coomassie brilliant blue R-250.

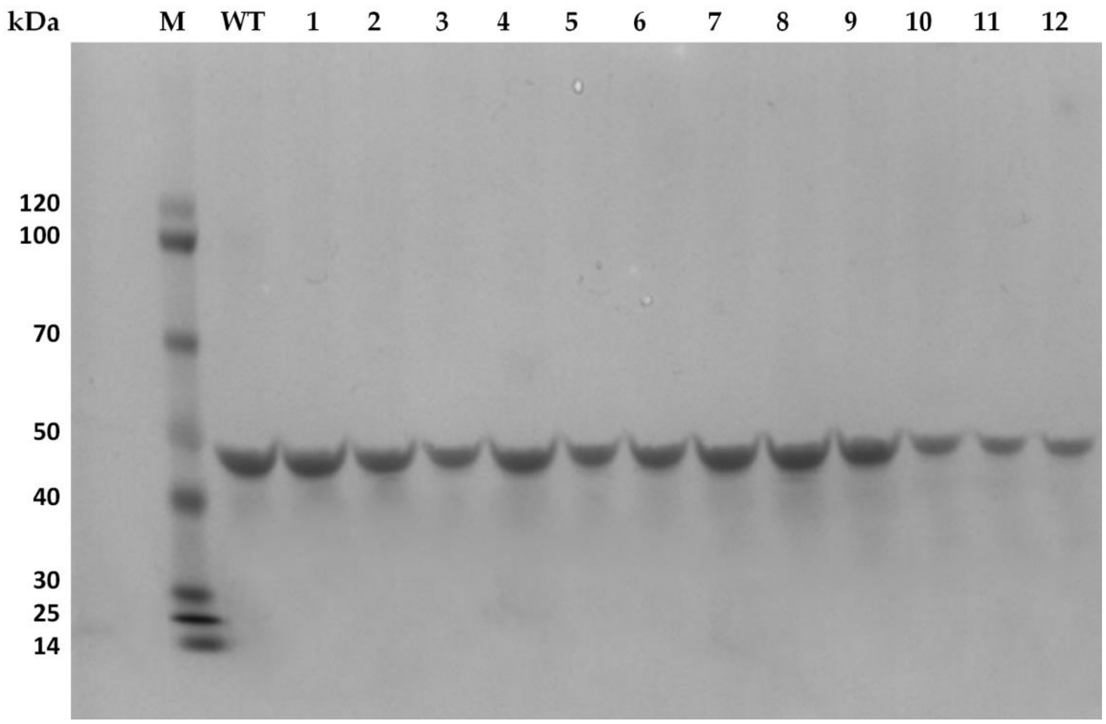

Figure S2. SDS-PAGE (12%) analysis of OYE3 and 12 purified OYE3 variants with single substitution at site W116 or S296. Lane M, standard molecular mass proteins; lane WT, wild type OYE3. Other lanes from 1 to 12 represent the OYE3 variants (from left to right): S296F, S296W, S296Y, S296A, S296G, W116A, W116V, W116I, W116F, W116Y, W116S and W116G. The proteins were visualized by staining with Coomassie brilliant blue R-250.

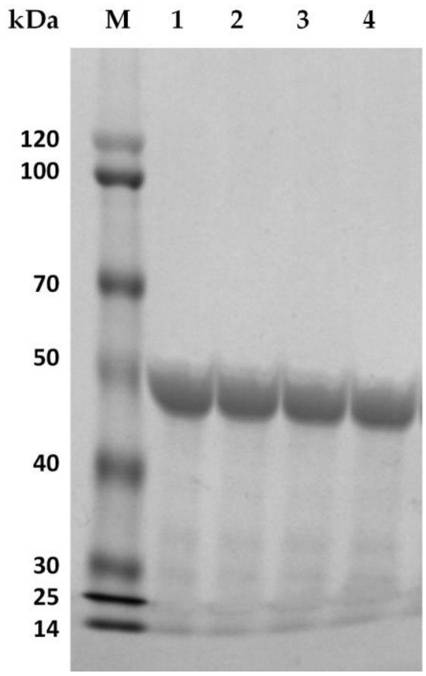

Figure S3. SDS-PAGE (12%) analysis of 4 purified OYE3 variants with double substitution at sites W116 and S296. Lane M, standard molecular mass proteins; lane WT, wild type OYE3. Other lanes from 1 to 4 represent the OYE3 variants (from left to right): S296F/W116V, S296F/W116S, S296F/W116A and S296F/W116G. The proteins were visualized by staining with Coomassie brilliant blue R-250.

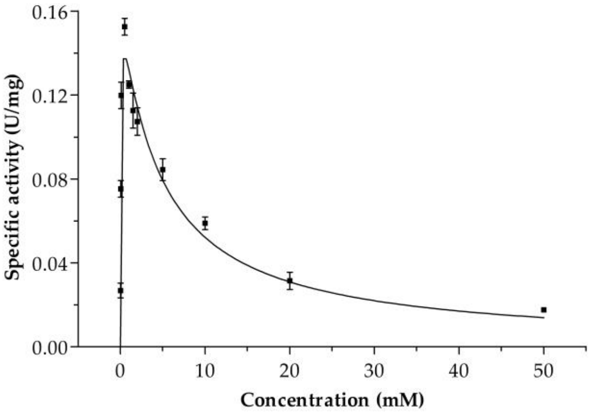

(a)

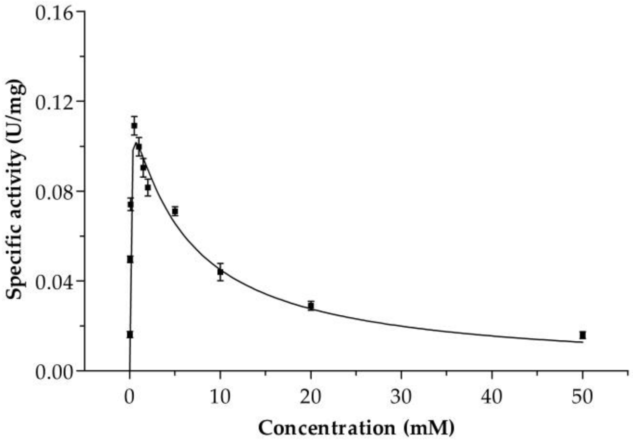

(b)

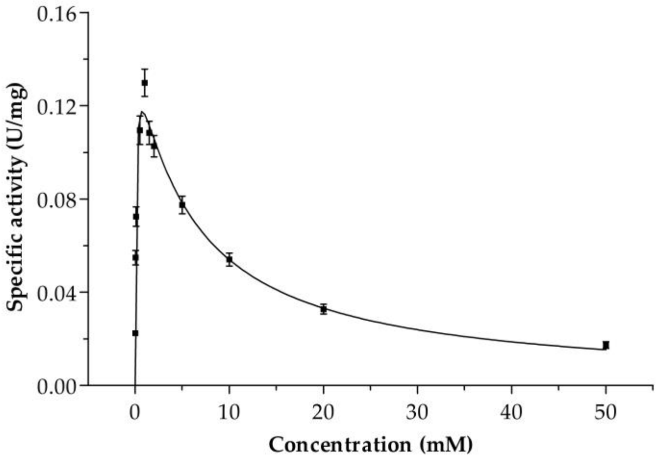

(c)

Figure S4. Apparent kinetic parameter determination of OYE3 and its variant S296F/W116G. According to substrate-inhibition kinetics, apparent values of K_m, K_i and V_{max} were calculated using the curve fittings of the software Prism 7.2 (GraphPad Software, San Diego, CA).

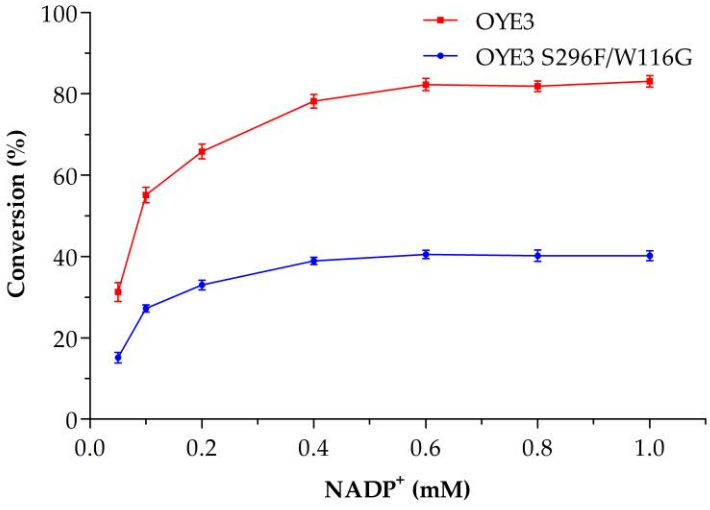

(a)

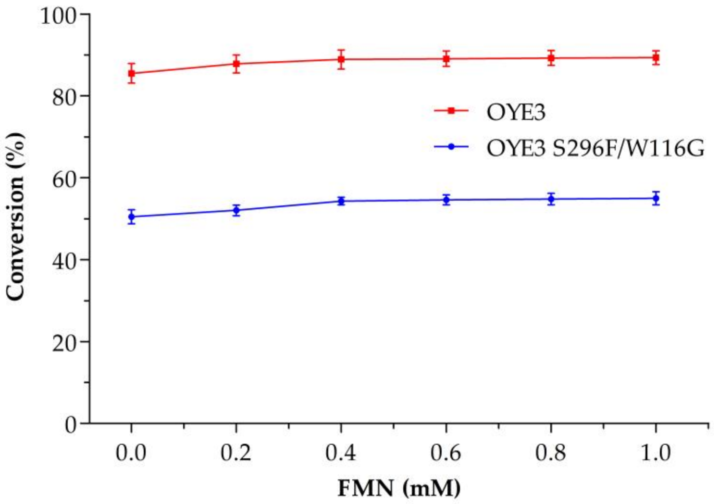

(b)

Figure S5. Effect of $NADP^+$ (a) and FMN (b) on the reduction of (*E*/*Z*)-citral to (*R*)-citronellal. The standard reaction mixture (10 mL) contained 20 mM (*E*/*Z*)-citral, $NADP^+$, 0.15 U/mL GDH, 50 mM glucose, 0.15 U/mL of the variant S296F/W116G, and 50 mM PIPES buffer solution (pH 7.0). The effect of $NADP^+$ concentration were investigated from 0 to 1 mM. When the $NADP^+$ concentration was fixed at 0.4 mM, the FMN concentrations were investigated from 0 to 1 mM. The stock solution of substrate was 200 mM citral dissolved in isopropanol. The reaction was conducted in a reactor with a pH auto-titration system at 30 °C and 400 rpm for 10 h (OYE3) or 18 h (the variant S296F/W116G). Data present mean values ± SD from three independent experiments. (*E*/*Z*)-Citral contained 58.4% geranial and 41.6% neral.

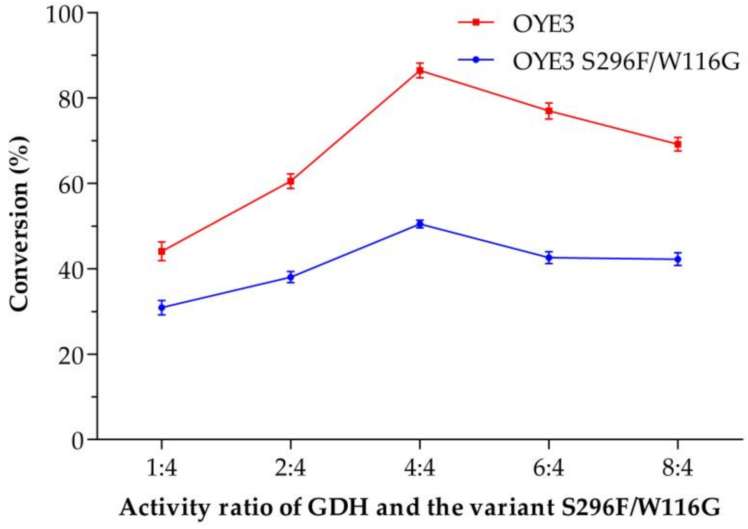

(a)

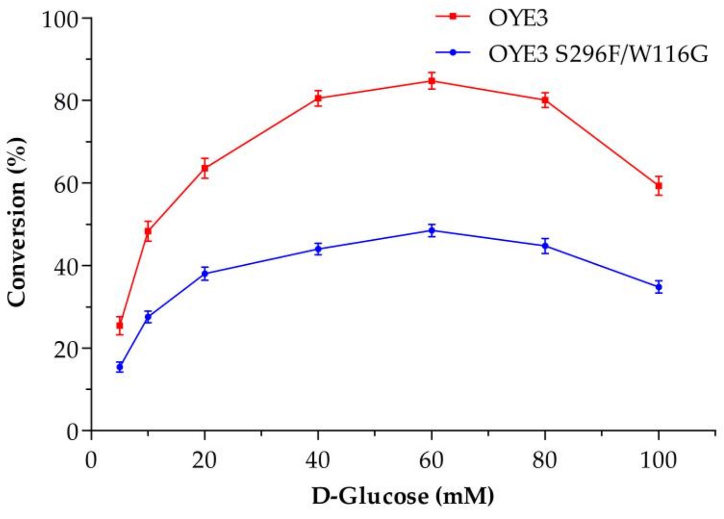

(b)

Figure S6. Effect of glucose dehydrgenase (a) and glucose (b) on the reduction of (*E*/*Z*)-citral to (*R*)-citronellal. The standard reaction mixture (10 mL) contained 20 mM (*E*/*Z*)-citral, 0.4 mM $NADP^+$, GDH, 50 mM glucose, 0.15 U/mL of the variant S296F/W116G, and 50 mM PIPES buffer solution (pH 7.0). The investigated ratios of GDH and the variant S296F/W116G included 1: 4, 2: 4, 4: 4, 6: 4 and 8: 4 when the variant S296F/W116G and glucose were fixed at 0.15 U/mL and 50 mM, respectively. When the equal activity ratio of GDH and the variant S296F/W116G was used, the glucose concentrations were investigated from 5 to 100 mM. The stock solution of substrate was 200 mM citral dissolved in isopropanol. The reaction was conducted in a reactor with a pH auto-titration system at 30 °C and 400 rpm for 10 h (OYE3) or 18 h (the variant S296F/W116G). Data present mean values ± SD from three independent experiments. (*E*/*Z*)-Citral contained 58.4% geranial and 41.6% neral.

>OYE3
ATGCCGTTTGTTAAAGGGTTTGAACCGATTAGCCTGCGTGACACCAACCTGTTCGAAC
CGATTAAAATTGGGAACACCCAGCTGGCCCACCGTGCCGTTATGCCGCCGCTGACCCG
CATGCGTGCAACCCACCCGGGTAATATTCCGAATAAAGAATGGGCAGCAGTTTATTAT
GGTCAGCGCGCACAGCGTCCGGGTACTATGATTATTACAGAGGGCACATTTATTTCACC
GCAGGCAGGAGGCTATGATAATGCGCCGGGTATCTGGTCAGATGAACAGGTGGCAGA
GTGGAAAAACATCTTTCTGGCAATTCACGACTGTCAGAGCTTTGCATGGGTCCAGCTG
TGGAGTCTGGGTTGGGCGAGTTTTCCGGATGTTCTGGCGCGTGATGGGCTGCGTTATGA
TTGTGCAAGCGATCGTGTTTATATGAATGCAACGCTGCAGGAGAAAGCGAAAGATGC
AAATAATCTGGAGCATAGCCTGACCAAAGATGATATTAAACAGTATATCAAAGACTAC
ATCCACGCAGCAAAAAATAGCATTGCAGCAGGTGCAGATGGTGTTGAAATTCATAGC
GCAAATGGTTATCTGCTGAATCAGTTTCTGGATCCGCATAGCAATAAACGTACCGATGA
ATATGGTGGTACCATTGAAAATCGTGCACGTTTTACCCTGGAAGTTGTTGATGCACTGA
TTGAAACCATTGGTCCGGAACGTGTTGGTCTGCGTCTGAGCCCGTATGGTACCTTTAAT
AGCATGAGCGGTGGTGCAGAACCGGGTATTATTGCACAGTATAGCTATGTTCTGGGTG
AACTGGAAAAACGTGCAAAAGCAGGTAAACGTCTGGCATTTGTTCATCTGGTTGAAC
CGCGTGTTACCGATCCGAGCCTGGTTGAAGGTGAAGGTGAATATAGCGAAGGTACCA
ATGATTTTGCATATAGCATTTGGAAAGGTCCGATTATTCGTGCAGGTAATTATGCACTGC
ATCCGGAAGTTGTTCGTGAACAGGTTAAAGATCCGCGTACCCTGATTGGTTATGGTCGT
TTTTTTATTAGCAATCCGGATCTGGTTTATCGTCTGGAAGAAGGTCTGCCGCTGAATAA
ATATGATCGTAGCACCTTTTATACCATGAGCGCAGAAGGTTATACCGATTATCCGACCT
ATGAAGAAGCAGTTGATCTGGGTTGGAATAAAAATTAA

>GDH
ATGGGTTATAATTCTCTGAAAGGCAAAGTCGCGATTGTTACTGGTGGTAGCATGGGCAT
TGGCGAAGCGATCATCCGTCGCTATGCAGAAGAAGGCATGCGCGTTGTTATCAACTAT
CGTAGCCATCCGGAGGAAGCCAAAAAGATCGCCGAAGATATTAAACAGGCAGGTGGT
GAAGCCCTGACCGTCCAGGGTGACGTTTCTAAAGAGGAAGACATGATCAACCTGGTG
AAACAGACTGTTGATCACTTCGGTCAGCTGGACGTCTTTGTGAACAACGCTGGCGTTG
AGATGCCTTCTCCGTCCCACGAAATGTCCCTGGAAGACTGGCAGAAAGTGATCGATGT
TAATCTGACGGGTGCGTTCCTGGGCGCTCGTGAAGCTCTGAAATACTTCGTTGAACAT
AACGTGAAAGGCAACATTATCAATATGTCTAGCGTCCACGAAATCATCCCGTGGCCTA
CTTTCGTACATTACGCTGCTTCTAAGGGTGGCGTTAAACTGATGACCCAGACTCTGGCT
ATGGAATATGCACCGAAAGGTATCCGCATTAACGCTATCGGTCCAGGCGCGATCAACA
CTCCAATTAATGCAGAAAAATTCGAGGATCCGAAACAGCGTGCAGACGTGGAAAGCA
TGATCCCGATGGGCAACATCGGCAAGCCAGAGGAGATTTCCGCTGTCGCGGCATGGC
TGGCTTCTGACGAAGCGTCTTACGTTACCGGCATCACCCTGTTCGCAGATGGTGGCAT
GACCCTGTACCCGAGCTTTCAGGCTGGCCGTGGTTGA

Figure S7. The codon-optimized nucleotide sequences encoding old yellow enzyme OYE3 from *Saccharomyces cerevisiae* S288C and glucose dehydrogenase from *Exiguobacterium sibiricum*.

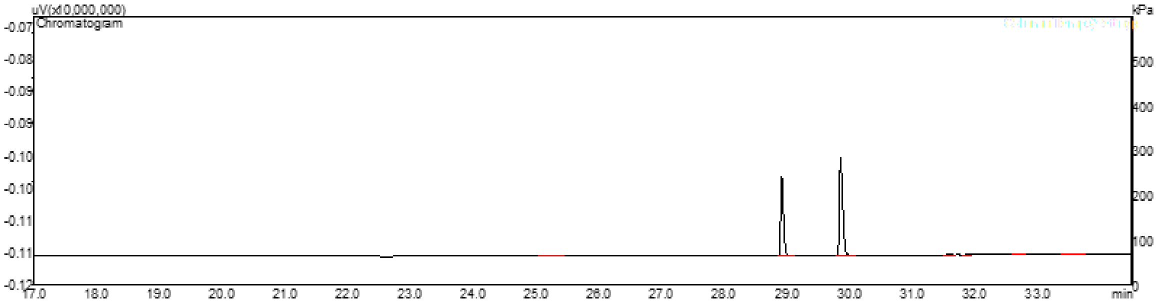

(a)

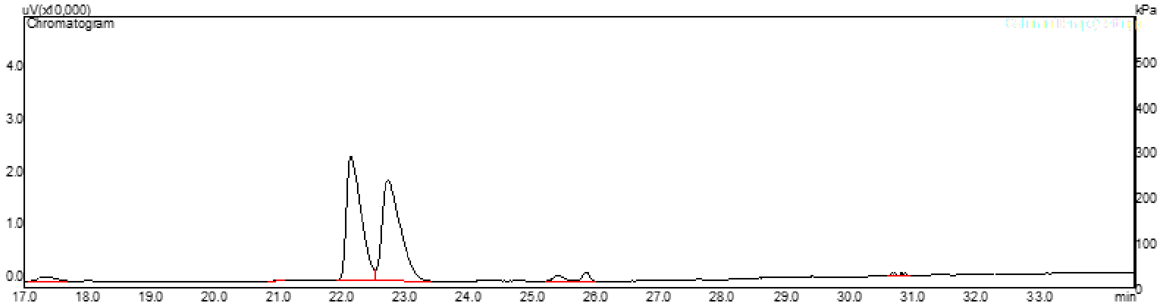

(b)

Figure S8. The GC chromatogram of standard substrates (a) and standard products (b). The retention times of (*S*)-citronellal, (*R*)-citronellal, (*Z*)-citral and (*E*)-citral were 22.5 min, 23.0 min, 29.2 min and 30.2 min.
